# Placenta Prœvia

**Published:** 1856-05

**Authors:** 


*Placenta Prævia.*—Dr. Barnes has proposed before the Medical Society of London, a new method of procedure in this accident—or rather a revival of a method previously proposed. The new operation consists in: 1. Determining the side of the uterus; 2. In rupturing the membranes and detaching the placenta from this half of its circumference; 3. Exciting uterine contraction; 4. Hooking finger over edge of placenta, tearing the membranes from the freed border of the placenta; and 5. In separating the placenta in a circumference of 190 to 200 degrees.

